# Measuring mindfulness in children: breath counting is unrelated to self-reported mindfulness but improves after mindfulness practice in 9–13 year-olds

**DOI:** 10.3389/fpsyg.2025.1644127

**Published:** 2025-09-30

**Authors:** Winnie Zhuang, Laura E. Michaelson, Sona Dimidjian, Yuko Munakata

**Affiliations:** 1 Renée Crown Wellness Institute, University of Colorado Boulder, Boulder, CO, United States; 2 Department of Psychology and Center for Mind and Brain, University of California, Davis, Davis, CA, United States; 3 American Institutes for Research, Arlington, VA, United States; 4 Department of Psychology and Neuroscience, University of Colorado Boulder, Boulder, CO, United States

**Keywords:** mindfulness, self-report, breath counting task, cognitive control, children

## Abstract

Recent calls for mindfulness measures beyond self-report abound, especially for children. Because breath awareness is central to many mindfulness practices, the breath counting task has been proposed as a behavioral measure of mindfulness for adults. In the current study, we investigated whether the breath counting task can also serve as a valid behavioral measure of children’s mindfulness. We examined psychometric properties across breath counting, three established mindfulness questionnaires, and a behavioral cognitive control measure in a sample of 109 children ages 9–13 years. We also offered 1–2 weeks of audio-based mindfulness training to a subset of children (*n =* 67) who completed daily breathing exercises, then reassessed their breath counting and self-reported mindfulness. In the full sample, children’s breath counting showed psychometric properties and patterns similar to those of adults, was positively associated with overall cognitive control performance, but was unrelated to their self-reported mindfulness (*p*’s > 0.24). However, breath counting did improve following training amongst the subset of children who completed 1–2 weeks of daily mindfulness exercises (*p* < 0.001, *η*^2^ = 0.23), whereas self-reported mindfulness did not (*p* = 0.44). Our findings suggest that the breath counting task captures aspects of mindfulness separate from those measured by children’s self-reports, and may be more sensitive to training impacts. We recommend the use of both self-report and behavioral measures of mindfulness, like the breath counting task, in future work.

## 1 Introduction

Mindfulness-based exercises and meditation have become popular techniques to support psychological wellbeing in children. Yet impacts of mindfulness programs, which aim to cultivate present-moment awareness and a non-judgmental attitude, have been mixed. Some systematic reviews report small to moderate positive effects of mindfulness on children’s mental health and wellbeing (Carsley et al., 2018; Dunning et al., 2019), while others indicate inconsistent effects across outcome domains and study designs (Fulambarkar et al., 2023; Odgers et al., 2020). Additionally, the field of mindfulness research has struggled to reliably and objectively define and measure mindfulness, especially in children (Chems-Maarif et al., 2025; Goodman et al., 2017). Usually self-reported questionnaires are used, but these types of measures rely on accurate participant self-assessment and may be prone to various biases (Bergomi et al., 2013). These issues are especially relevant in children, whose still-developing metacognitive skills may hinder accurate self-assessment and whose reliance on adults may make them especially keen to respond in ways that are socially-pleasing (Camerini and Schulz, 2018; Miller et al., 2015). Thus, calls for more objective mindfulness measures for children and youth abound (Goodman et al., 2017; Felver et al., 2018; Davidson and Kaszniak, 2015).

To address the shortcomings of self-reports, several behavioral assessments have been proposed to measure mindfulness in adults (see Hadash and Bernstein, 2019, for a review). One of these is the breath counting task, an assessment inspired by breath awareness exercises that are common in many mindfulness programs (Simkin and Black, 2014). In the breath counting task, participants count their breaths in sets of nine over the course of several minutes; participants’ breath counting accuracy can be determined as the proportion of correctly counted breath sets. In its validation study, adults’ breath counting accuracy was correlated with self-reported mindfulness and with task-based sustained attention (Levinson et al., 2014). These results were replicated in later studies, and additional scores, namely miscounts and resets, were proposed to reflect different aspects of attention (Wong et al., 2018; Ching and Lim, 2023). More recently, breath counting performance was found to better differentiate between individuals who completed an 8-week mindfulness training from active controls than self-reports (Isbel et al., 2020), suggesting that the breath counting task more accurately captures gains from mindfulness training. Overall, the breath counting task has shown some convergent validity with self-reported mindfulness in adults and may also capture attentional aspects of mindfulness that are not fully accounted for by self-reports.

There are several reasons to hypothesize that the breath counting task may also offer a suitable alternative to self-report measures of mindfulness for children. First, procedural counting, the numeracy skill engaged during breath counting, is an age-appropriate task that is not difficult for even very young children to do (Litkowski et al., 2020). Second, breath awareness training is common across mindfulness-based programs for children (Vekety et al., 2022); as such, breath counting accuracy may serve as an appropriate metric to distinguish children with different levels of mindfulness. Finally, the breath counting task may offer a way to circumnavigate issues of bias in self-reports. As noted previously, self-reported measures of mindfulness may be influenced by social desirability bias (Camerini and Schulz, 2018) and inaccurate self-reporting—these factors may distort or even inflate children’s self-reported mindfulness scores (Goodman et al., 2017; Roux et al., 2019). The breath counting task may offer a reliable alternative because its performance-based scores are designed to capture behavior objectively rather than through self-assessment (Levinson et al., 2014).

To our knowledge, no studies have yet examined the applicability of the breath counting task as a measure of mindfulness for children. Some studies have explored breath counting in adolescents but reveal mixed findings. In a small sample of adolescents who engaged in a meditative exercise that involved breath counting, greater “blissfulness” experienced during breath counting was shown to be associated with greater state mindfulness (Brahmi et al., 2025). Conversely, in a convenience sample of adolescents with elevated rumination, breath counting performance did not correlate with self-reported mindfulness and other relevant mental health measures (Treves et al., 2025). These mixed findings in adolescents may be attributable to unique characteristics of their samples, and the use of distinct measures of mindfulness. Given the potential for bias in self-reports, adolescents’ self-reported mindfulness may not have accurately measured their mindfulness and thus led to the unexpected results. Alternatively, breath counting performance may capture aspects of mindfulness that are missed by self-reports alone. The degree to which the breath counting task measures mindfulness in a general, non-clinical sample of children remains unknown.

Furthermore, the breath counting task may provide an avenue to identify some of the cognitive mechanisms underlying mindfulness. One candidate mechanism proposed to be relevant for mindfulness is cognitive control, the ability to overcome a prepotent behavior and successfully execute a goal-directed action. Researchers have long speculated that cognitive control, and executive functions more broadly, are involved in bidirectional relationships with mindfulness (Malinowski, 2013). Emerging evidence using the breath counting task shows some support for this claim: in adults, breath counting performance was found to predict better performance in the sustained attention to response task (SART; Levinson et al., 2014) and fewer attentional lapses during a psychomotor vigilance task (Wong et al., 2018), both behavioral measures of sustained attention and closely related to cognitive control (Hilti et al., 2013). This result was partially replicated in ruminative adolescents as well (Treves et al., 2025), suggesting that cognitive control may play a role in mindfulness among youth.

Studies have also begun unraveling changes in preferred modes of cognitive control after mindfulness practice. A prominent guiding framework in the cognitive control literature is the dual mechanisms of control (DMC; Braver, 2012), which posits that cognitive control can be distinguished into two modes based on its temporal dynamics: reactive control (engaging control in the moment, *as needed*) and proactive control (engaging control in preparation, *before* it is needed). A rich literature has and continues to explore the inter- and intra-individual factors that shape preferences for reactive or proactive control (e.g., Smittenaar et al., 2015; Yang et al., 2018), and researchers largely agree that adaptively engaging both forms of control is important for successful achievement of goals and general functioning (Braver, 2012; Chevalier et al., 2020; Mäki-Marttunen et al., 2019). However, the nature of associations between mindfulness and cognitive control modes remains unclear. One study with a small sample size (*N =* 30) found that after an 8-week mindfulness training program, adults demonstrated higher levels of proactive control compared to a control group (Li et al., 2018). However, in at least two other studies with larger sample sizes, adults higher in self-reported mindfulness showed more balanced, flexible engagement of both proactive and reactive control strategies compared to adults lower in self-reported mindfulness, who tended to engage control more proactively (Chang et al., 2018; Aguerre et al., 2021). The latter findings suggest that mindfulness allows individuals to engage present-moment awareness and respond adaptively to contextual demands on cognitive control, shifting control strategies towards more balanced modes.

It remains an open question how mindfulness and cognitive control are associated in children. Several studies and even meta-analyses have offered compelling evidence that mindfulness training improves executive functions related to cognitive control in children (Mak et al., 2018; Takacs and Kassai, 2019; Bauer et al., 2020). However, these studies primarily rely on self-report measures of mindfulness and thus are subject to all the problems of self-report described above. Further, to our knowledge, no study has directly explored the relationship between children’s mindfulness and their cognitive control per se. Cognitive control develops gradually across childhood (Crone and Steinbeis, 2017; Davidson et al., 2006)—for example, young children predominantly prefer reactive control, showing a transition to increasingly more proactive control around 5–6 years, and thereafter become more adaptive and flexible in their use of both forms control (Munakata et al., 2012; Lucenet and Blaye, 2014; Troller-Renfree et al., 2020). Mindfulness may support this development by supporting the emergence of proactive control in young children, and encouraging more flexible use of proactive and reactive control in older children. Further, these associations may be more clearly detected using more direct measures of mindfulness and cognitive control. In the current study, we used the breath counting task, self-reported mindfulness, and a performance-based measure of cognitive control to examine the relationships between children’s mindfulness and cognitive control. Unraveling these relationships will provide insights into cognitive control’s role as a mechanism underlying mindfulness in children.

The current study seeks to examine the validity of the breath counting task as a measure of mindfulness in children ages 9–13 years and its associations with cognitive control. We first examined children’s performance patterns and the psychometric properties of the breath counting task’s three indices: accuracy, miscounts, and resets. Next, we examined the breath counting task’s convergent validity with self-report measures of mindfulness, controlling for social desirability bias. We predicted that self-reported mindfulness would show significant positive correlations with children’s breath counting performance indices after controlling for social desirability bias in self-reports. Then, we explored relationships between children’s breath counting performance and their cognitive control measured using the cued task switching paradigm, a commonly used task to assess cognitive control in children and adults (Cepeda et al., 2001). We predicted that children with better breath counting performance would show a stronger preference for proactive control when it is made possible compared to when it is made impossible in the task, reflecting an adaptive engagement of cognitive control. Finally, we examined changes in children’s mindfulness as assessed by the breath counting task and by self-report after 1–2 weeks of daily mindfulness practice. We predicted that after mindfulness practice, children’s breath counting indices would improve while their self-reported mindfulness scores would not change, reflecting a greater sensitivity of the breath counting task to immediate changes in mindfulness relative to self-reports. These findings will shed light on the suitability of the breath counting task as a mindfulness measure for children and the degree to which cognitive control is associated with children’s mindfulness.

## 2 Materials and methods

### 2.1 Participants

A sample of 109 children ages 9–13 years (*M =* 11.64 years, SD *=* 1.11, range = 9.08–12.9; *n* males = 61, females = 48, other = 0) were recruited to participate in this study. Our sample size was determined based on practical constraints (time and monetary) as specified in our preregistration on Open Science Framework^1^. Participants were recruited through advertisements, community tabling, and from a database of families who expressed interest in participating in developmental research in the Boulder, CO, USA area. All participant demographics are described in Table 1. The racial composition of our sample was 78.0% White, 2.8% Asian, 5.5% mixed race, and 13.7% unreported. The ethnic composition of our sample was 74.3% non-Hispanic, 8.3% Hispanic or Latino, and 17.4% unreported. Written informed consent was obtained from a parent or legal guardian, and written child assent was obtained prior to participation. Parents or legal guardians received monetary compensation for time and travel, and children chose tokens (e.g., small toys, stuffed animals) and/or a book for their participation. The local Institutional Review Board approved all study procedures (Protocol #18-0060).

**Table 1:** Participant demographics.

Demographic characteristic	*N*	%
Gender		
Female	48	44.03
Male	61	55.96
Race		
White	85	78.00
Asian or Asian American^a^	3	2.80
Mixed Race	6	5.50
Unreported	15	13.70
Ethnicity		
Hispanic or Latino	9	8.30
Not Hispanic or Latino	81	74.30
Unreported	19	17.40

Parents’ highest education level	Parent 1		Parent 2	
4-year college or more	64	58.72	52	47.71
HS graduate, some college	2	1.83	6	5.50
HS graduate, no college	0	0.00	2	1.83
Unreported	43	39.45	49	44.95

A total of 109 participants were included in this study.

a We acknowledge that it can be problematic to group Asian and Asian American into a single race category, as this confounds individuals of Asian ancestry originating from Asia with those who originate from America (or elsewhere; APA, 2015). Unfortunately, our questionnaire did not make the distinction between Asians and Asian Americans (or Asians of other places of origin), preventing us from accurately identifying separate Asian-identifying groups in our sample. Future studies should separate these groups to more accurately reflect the diversity of individuals with Asian ancestry (see APA’s JARS-REC for detailed guidelines).

### 2.2 Design and procedure

This study was designed as a cross-sectional study with an optional intervention. All participants completed an initial 2-h laboratory session, and participants who opted to participate in a 1–2-week mindfulness intervention also completed a second, 1-h laboratory session 1–2 weeks after the first laboratory session. Thus, the intervention in this study was implemented using a single group pre-post design.

During the first 2-h laboratory session, children completed a battery of questionnaires and computerized tasks that were part of a larger study examining measures of mindfulness and impacts of weeklong daily mindfulness practice. The laboratory session took place in a quiet testing room with the experimenter present at all times. Children’s parents remained in the lab waiting area and completed parent-report questionnaires during the testing session.

In the testing room, mindfulness questionnaires were administered at the start of the session. To begin, the experimenter and the participant sat at opposite ends of a table. The participant was provided a clipboard with the printed questionnaires and a pen. The experimenter then proceeded through each questionnaire, reading aloud each item, and the participant was instructed to mark their responses on the printed questionnaire out of the experimenter’s vantage point. The experimenter encouraged the participant to answer as best they could, welcomed any clarification questions, and noted that all responses were fully confidential. After each questionnaire, the participant was instructed to place their completed forms inside a folder on the table so that the experimenter could not view their responses. These measures were implemented to reduce direct impacts of social desirability bias while the participant completed self-reports. The questionnaires were administered in the same order for all participants: first the Mindfulness Inventory for Children and Adolescents (MICA), then the Mindful Attention Awareness Scale-Children (MAAS-C), and finally the Child and Adolescent Mindfulness Measure (CAMM). This segment of the study took approximately 20 min.

Next, participants proceeded to a set of computerized behavioral and physiological tasks, including the breath counting task and the cued task switching paradigm (CTS), a performance-based measure of cognitive control. All behavioral and physiological tasks were administered on a computer with the child seated roughly 60 cm from the screen, and children provided responses using the keyboard. The experimenter introduced each task to the child and remained present in the testing room at all times. The full set of computerized tasks took approximately 1 h to complete. Children were encouraged to take breaks between tasks as needed.

During the breath counting task, physiological measures were recorded using Alive Pioneer with Gp8 Amp (Somatic Vision©). Children were asked to wear a breathing belt on top of their clothing around their chest or waist to measure their respiratory rate. Two other sensors, placed on the middle finger to measure blood pulse volume heart rate and on the pointer and ring fingers to measure skin conductance, were also worn for purposes unrelated to the current investigation. An experimenter explained each sensor and its purpose before asking children to wear the items. Children were given a demonstration of each sensor using the Alive Pioneer Graph Training application (Encinitas, Somatic Vision©). Following the demonstration, the monitor was turned off so that children were seated in front of a black monitor. Children were asked to minimize movement and talking while wearing the sensors. Physiological outputs were monitored during the tasks by the experimenter using a computer situated behind the participant. Children then proceeded to complete the breath counting task (described in detail in the Measures section below) followed by two other physiological awareness tasks unrelated to the current investigation.

After completion of behavioral and physiological tasks, children completed the social desirability scale in the same manner as the mindfulness questionnaires. Afterwards, children viewed an informational video introducing them to a set of optional, at-home mindfulness activities that they could elect to complete. Children were given the option to participate in a second lab session, scheduled one to 2 weeks from their first session, if they opted to complete the daily at-home mindfulness activities. Finally, children and families were thanked and gifted with a prize token and $15 for their participation in the first session.

After the first session, children who opted to participate in the second session were asked to complete daily at-home mindfulness activities for 1–2 weeks. These daily mindfulness activities consisted of seven unique 5- to 10-min guided audio breath focused exercises. Each exercise focused on a different theme, including noticing, attending, counting, and slowing down one’s breath. The exercises were selected from a library of proprietary contemplative sessions developed for children; all exercises focused on practicing breath-focused awareness and attention. Each day during the 1–2 week period, unique links for each of the seven audio-guided exercises were sent via email to children’s parents, who were instructed to provide the link to their child. Children were asked to complete each exercise uninterrupted, by themselves, in a quiet area. To verify exercise completion and engagement, we checked that the time individuals spent on the linked web page containing the audio exercise equaled the length of the audio exercise, and asked children to write a short reflection of their experiences after each daily exercise. If children did not complete the full duration of an exercise, a link to the incomplete exercise was resent the following day; links for a given exercise were resent until completed or up to five times after which no additional links were sent.

To ensure that children experienced the full set of seven audio exercises, we required children to have completed all seven exercises at least once before returning for their second session. This requirement was only implemented partway through data collection and thus a small number of children (*n* = 15) completed fewer than seven mindfulness activities in the final sample of children who returned for the second session (*n =* 67).

After completing all seven exercises, children were invited to return for a second laboratory session. The second session followed a similar format to the first with an abridged set of measures administered. Children completed a measure of self-reported mindfulness (the MICA only), the breath counting task, and the CTS as before. Additional measures were also administered in the second session that are not part of the current investigation. Children and their parents received additional monetary compensation and prize tokens for participating in the second session.

### 2.3 Measures

#### 2.3.1 Mindfulness questionnaires

##### 2.3.1.1 Child and adolescent mindfulness measure (CAMM)

The Child and Adolescent Mindfulness Measure (CAMM; Greco et al., 2011) is a validated 10-item survey that assesses mindfulness skills in school-aged children and adolescents. Items include statements such as “I get upset with myself for having feelings that do not make sense” or “I push away thoughts that I do not like.” Participants rate each item on a 5-point Likert scale where 0 = Never True and 4 = Always True. Summary scores are computed by first reverse scoring each item and then averaging across items, with higher scores reflective of better mindfulness skills. The CAMM exhibited a one-factor solution and reliable internal consistency (Cronbach’s alpha = 0.80) in previous work with 10–17-year-olds (Greco et al., 2011) and in our sample (Cronbach’s alpha = 0.80).

##### 2.3.1.2 Mindful attention awareness scale-children (MAAS-C)

The Mindfulness Attention Awareness Scale for Children (MAAS-C; Lawlor et al., 2014) is a validated 15-item survey that assesses mindfulness in children ages 9–13. Participants rate statements indicative of low mindfulness, such as “I could be feeling a certain way and not realize it until later” and “I find myself doing things without paying attention,” on a 6-point Likert scale ranging from 1 = almost never to 6 = almost always. A summary score is computed by reverse scoring each item and then averaging, with higher scores reflective of more mindfulness. The MAAS-C was previously found to have a one-factor solution and to show reliable internal consistency (Cronbach’s alpha = 0.84); our sample also showed similarly good internal consistency (Cronbach’s alpha = 0.84).

##### 2.3.1.3 Mindfulness inventory for children and adolescents (MICA)

The Mindfulness Inventory for Children and Adolescents (MICA) is a self-report measure of children’s internal and external awareness and acceptance of thoughts and behaviors. Although not yet psychometrically evaluated in a published report, the instrument has demonstrated sensitivity to intervention impacts in an unpublished pilot study (Briere, 2011). This measure is designed to be developmentally appropriate for ages 8–18 and includes five theoretically-informed subscales (*self-acceptance, present-centered awareness, equanimity, metacognitive awareness,* and *acceptance of internal experiences*). Children respond to 25 items using a 5-point scale (1 = Disagree a lot, 5 = Agree a lot). Items included statements such as “I usually know what I am thinking or feeling” and “I like myself the way I am.” Because the MICA’s subscales have not yet been empirically validated at the writing of this manuscript, we opted to use the overall average MICA score, which exhibited adequate reliability (Cronbach’s alpha = 0.87) in our sample. Higher MICA scores indicate greater mindfulness.

#### 2.3.2 Children’s social desirability scale-short (CSDS)

Social desirability bias was measured using the Children’s Social Desirability Scale-Short form (CSDS; Miller et al., 2014). Children responded to 14 statements, including “Have you ever felt like saying unkind things to a person?” and “Do you always listen to your parents?” on a True and False response scale. Each item had a socially desirable response (e.g., “True” for “Do you always listen to your parents?”). This true-false response scale ensures that endorsement of the socially desirable response is most likely untruthful, because it is highly unlikely that a child has never “broken a rule” or never “gets mad when people do not do what you want them to do.” Thus, the CSDS’s response scale targets variance due to social desirability bias and not due to the specific contents of the items. A summary score was calculated by summing the number of socially desirable responses, with higher scores indicating more bias. The CDSC has demonstrated good internal consistency in previous work (Cronbach’s alpha = 0.84) and in our sample (Cronbach’s alpha = 0.77).

#### 2.3.3 Breath counting task (BCT)

The breath counting task has been validated as a behavioral index of dispositional mindfulness in adults (Levinson et al., 2014). In our child-adapted version of the task, children were instructed to be aware of their breath and count their breaths in sets of 9, silently in their head, over a fixed 6-minute interval. They indicated each set by pressing the down arrow on a keyboard for breaths 1–8 and the up arrow for breath 9, counting at the end of each exhale. Children were instructed to repeat the pattern after the 9th breath. Children were told if they lost count or made a mistake, they could press the spacebar to restart counting from breath 1. Thus, each set of breaths can be marked as accurate (eight down arrow presses followed by one up arrow press), a reset (any number of down arrow presses followed by one spacebar press), or a miscount (any non-eight number of down arrow presses followed by one up arrow press).

Children completed a 40-s practice block followed by a 6-min test block. At the start of both blocks, children heard a tone indicating that the block was about to begin, followed by the start tone to signal the start of counting. Children were instructed to keep counting until they heard the stop tone.

During this task, we also measured children’s actual breathing using respiratory inductive plethysmography with the respiration belt for GP8 Amp, Alive Pioneer, Somatic Vision©. A single respiratory belt was placed at the level of the abdomen and connected to a digital processing unit, which collected and outputted the raw respiratory signal. The Python package NeuroKit2 (Makowski et al., 2021) was used to process the raw respiratory signal and identify the occurrence of peaks (local maxima) and troughs (local minima) for each participant. We summed the number of peak-trough pairs to calculate the total number of breaths for each participant.

To process and score children’s breath counting performance, we followed procedures used with adults (Levinson et al., 2014). First, we verified that children’s counted breaths aligned with their measured breaths (as measured by the respiratory belt) by examining the correlation between the total number of breaths measured and the total number of key presses counted across participants. A high correlation would suggest that overall, children’s key presses matched their actual breath rates measured by the respiratory belt. Establishing this, we then proceeded to use the keypress data to calculate performance indices. To do so, each individual’s keypress data were split into breath sets based on the occurrence of an up arrow press (which indicates breath 9) or a spacebar press (which indicates a reset), both of which mark the end of a set. Each set then was categorized as an accurate set (a set that consisted of 8 down arrow presses followed by 1 up arrow press), a reset (a set that ended with a spacebar press), or a miscount (all other sets).

The primary index from the BCT was percentage accuracy, calculated as the number of accurate sets over the number of total sets. In addition to this primary index, we also calculated individuals’ percentage of resets and miscounts, which have been found to correlate with aspects of attention control in adults (Wong et al., 2018).

#### 2.3.4 Cued task switching (CTS) paradigm

The cued task-switching paradigm, adapted from Chevalier et al. (2015), was used to measure cognitive control. In this version of the task, children were asked to sort sea creatures based on a sorting cue (shape or color) displayed as shape or color icons in a border around the to-be-sorted sea creature. On each trial, children saw a fixation cross (1,000–1,200 ms), followed by a fishbowl (1,500 ms), and then the sea creature (target; 10 s or until response). The border remained on the screen through the entire trial.

Timing of the sorting cue differed between blocks in order to encourage proactive or reactive control. In the first block, the Proactive-Possible condition (Pro-Poss), the sorting cue was presented with the fishbowl *before* the target, allowing children to engage proactive control by maintaining the appropriate rule in advance. The cue remained visible following target onset, thus also enabling children to engage reactive control. In the second block, the Proactive-Impossible condition (Pro-Imp), the sorting cue was presented *with* the target at its onset, preventing proactive maintenance of the cue during the trial and requiring children to engage reactive control in order to successfully complete the task.

Before starting, children first practiced each sorting rule in sequence (four shape trials, four color trials), followed by mixed-rule practice (two shape, two color). During practice, accuracy feedback was provided (trial-level and summary feedback at end of the practice block) and incorrect trials were repeated. Children then completed a 16-trial practice block and reached an 80% accuracy rate before continuing. Children then completed the Pro-Poss condition followed by the Pro-Imp condition, with one test block per condition (39 trials). Conditions utilized different shape-color combinations: fish-shell-green-orange (Pro-Poss), crab-frog-red-blue (Pro-Imp) to reduce stimuli interference across blocks. Children responded using four buttons, one associated with each shape or color option, with response mapping reminders on screen during the task. Reaction times (RTs) and accuracy were measured for each trial.

Prior to data processing, we first verified that all individuals performed overall above chance (25%) in the CTS. Next, trials with RTs above 3 SDs from an individual’s mean were flagged as outliers and removed from analyses; this removed 1.43% of all trials. For RT analyses only, incorrect trials were excluded; this removed an additional 13.29% of trials for RT analyses. For comparisons between conditions, zRTs were computed by z-scoring RTs from each trial around each individual’s mean RT to account for differences in overall response speeds between individuals.

We focused on three target measures from this task: (1) mean accuracies and (2) median RTs from correct trials as measures of overall performance, and (3) the individually estimated effect of condition on z-scored RTs, or the zRT slope, which reflects the degree to which individuals engaged proactive control during the Pro-Poss compared to the Pro-Imp condition and served as a measure of children’s adaptive engagement of proactive control when made possible. A steeper, more negative zRT slope reflects faster responses during the Pro-Poss compared to the Pro-Imp condition, and thus more adaptive engagement of proactive control.

We also examined two exploratory measures from the CTS: (1) mean combined switch costs as a measure of task switching ability (Draheim et al., 2016), and (2) the total number of double errors as a measure of attentional lapses (Hajcak and Simons, 2008).

### 2.4 Data analysis plan

We first examined performance in the breath counting task and the reliability of its measures. Three participants who performed at or near zero accuracy appeared to have misunderstood or preferred not to comply with task instructions and were excluded from further analyses. Technical difficulties with the respiratory belt or the task set up led to the removal of 11 subjects. Thus, we were left with a final sample of 95 participants with complete data for the breath counting task analyses. With this final sample, we assessed relationships between breath counting indices and child-reported mindfulness scores to determine the convergent validity of breath counting as a measure of mindfulness. Then, we examined whether breath counting indices are related to children’s performance-based cognitive control. Finally, we assessed changes in children’s breath counting performance and self-reported mindfulness (MICA scores) after 1–2 weeks of at-home, daily mindfulness practice.

## 3 Results

### 3.1 Breath counting performance

**Figure 1:**
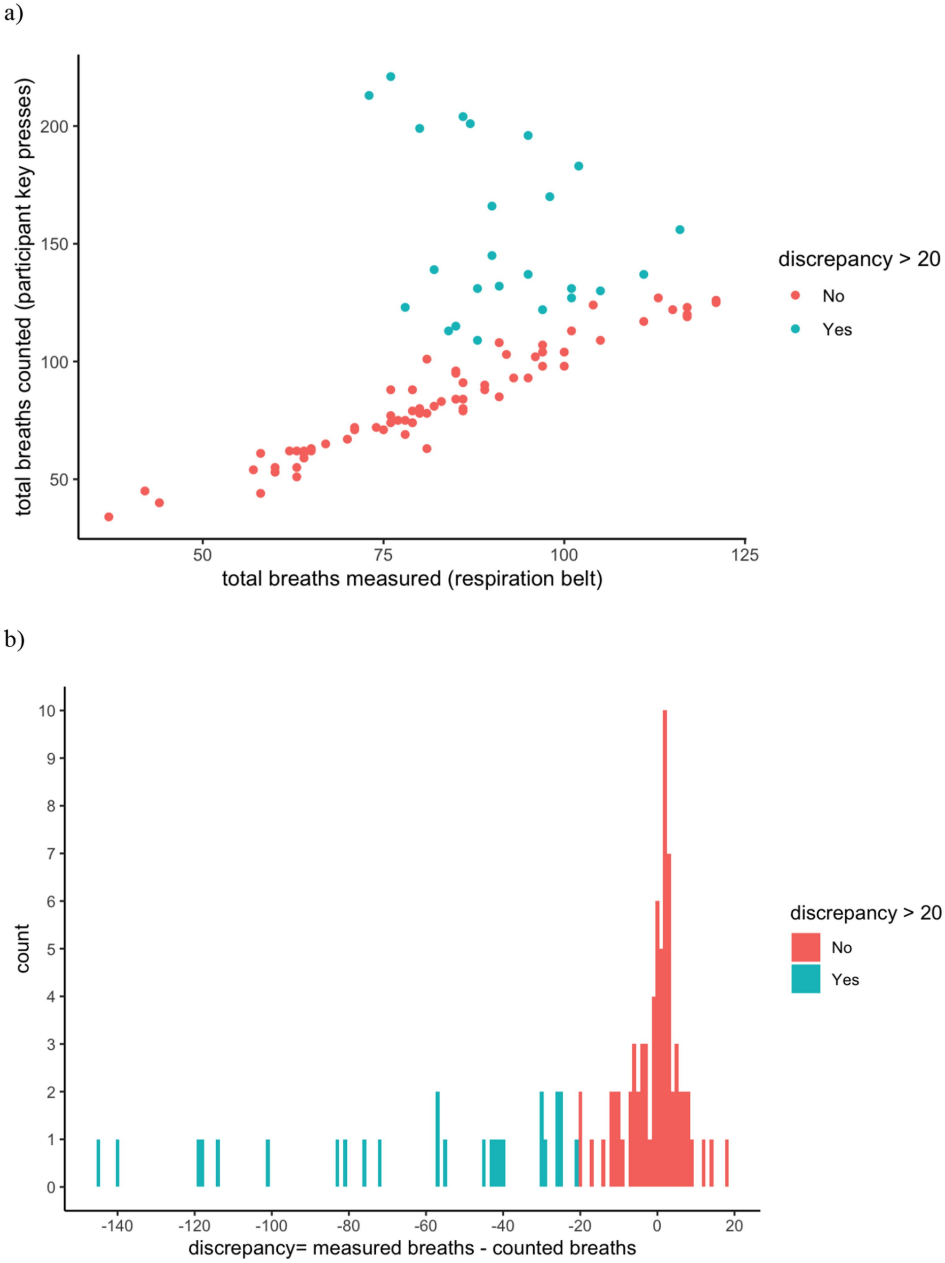
**(a)** Correlation between total breaths measured and counted. **(b)** Histogram of differences between total breaths measured and counted. The overall correlation between total breaths measured and counted was *r* = 0.575, *p* < 0.001 **(a)**, and the histogram of discrepancies between total breaths measured and counted showed a clear negative skew **(b)**. However, when participants with discrepancies >20 (blue, *n* = 24) were removed, the correlation between breaths counted and measured was *r* = 0.962, *p* < 0.001. All results and interpretations were consistent across analyses including and excluding the subset of participants with large discrepancies.

Descriptives from children’s breath counting task performance are shown in Table 2. First, we verified that children were counting their breaths (and not simply pressing the appropriate keys in sets of nine) during the breath counting task by examining the correlation between the total breaths measured (by the respiratory belt) and the total breaths counted (by key presses). The correlation was significant, *r* = 0.575, *p* < 0.001, but not as high as found in previous investigations with adults (e.g., *r* = 0.96 in Wong et al., 2018). A closer examination of the correlation plot revealed that 24 participants had a difference between their total breaths counted and total breaths measured of over 20 breaths (Figure 1a); further examination of these differences revealed a clear negatively skewed distribution, such that these discrepancies were driven by more breaths counted than measured (Figure 1b). Examination of the respiratory data from the 24 affected participants showed that discrepancies were likely due to poor signal quality arising from belt positioning and/or unnatural breathing, and exclusion of these 24 participants elevated the correlation between breaths counted and measured to *r* = 0.962, *p* < 0.001. We conducted all analyses with and without this group of individuals but all results and interpretations remained unchanged. Thus, in the interest of retaining maximal data and statistical power, we report our results based on the full sample of participants.

**Table 2:** Descriptives and internal reliabilities of measures from the breath counting task.

Variable	*M*	SD	Reliability^a^ 95% CI (LL, UL)	1	2	3	4	5	6
1. Age (years)	11.64	1.12	–						
2. Breaths counted (total)	100.95	40.88	–	−0.11					
3. Breaths measured (total)	84.53	17.67	–	−0.11	0.58**				
4. Sets counted (total)	12.01	4.6	–	−0.13	0.92**	0.54**			
5. Accuracy %	57.02	20.93	0.55 (0.4, 0.69)	0.18	0.1	0.14	0.02		
6. Resets %	11.19	11.95	0.54 (0.38, 0.68)	−0.01	−0.19	−0.21*	0.04	−0.34**	
7. Miscount %	31.78	20.28	0.39 (0.16, 0.57)	−0.18	0.01	−0.02	−0.04	−0.83**	−0.24*

*N* = 95; values displayed are Spearman-Brown corrected. *p < 0.05, **p < 0.01.

a Split half reliabilities were calculated from 5,000 random split-half permutations.

#### 3.1.1 Miscounts and resets

**Figure 2:**
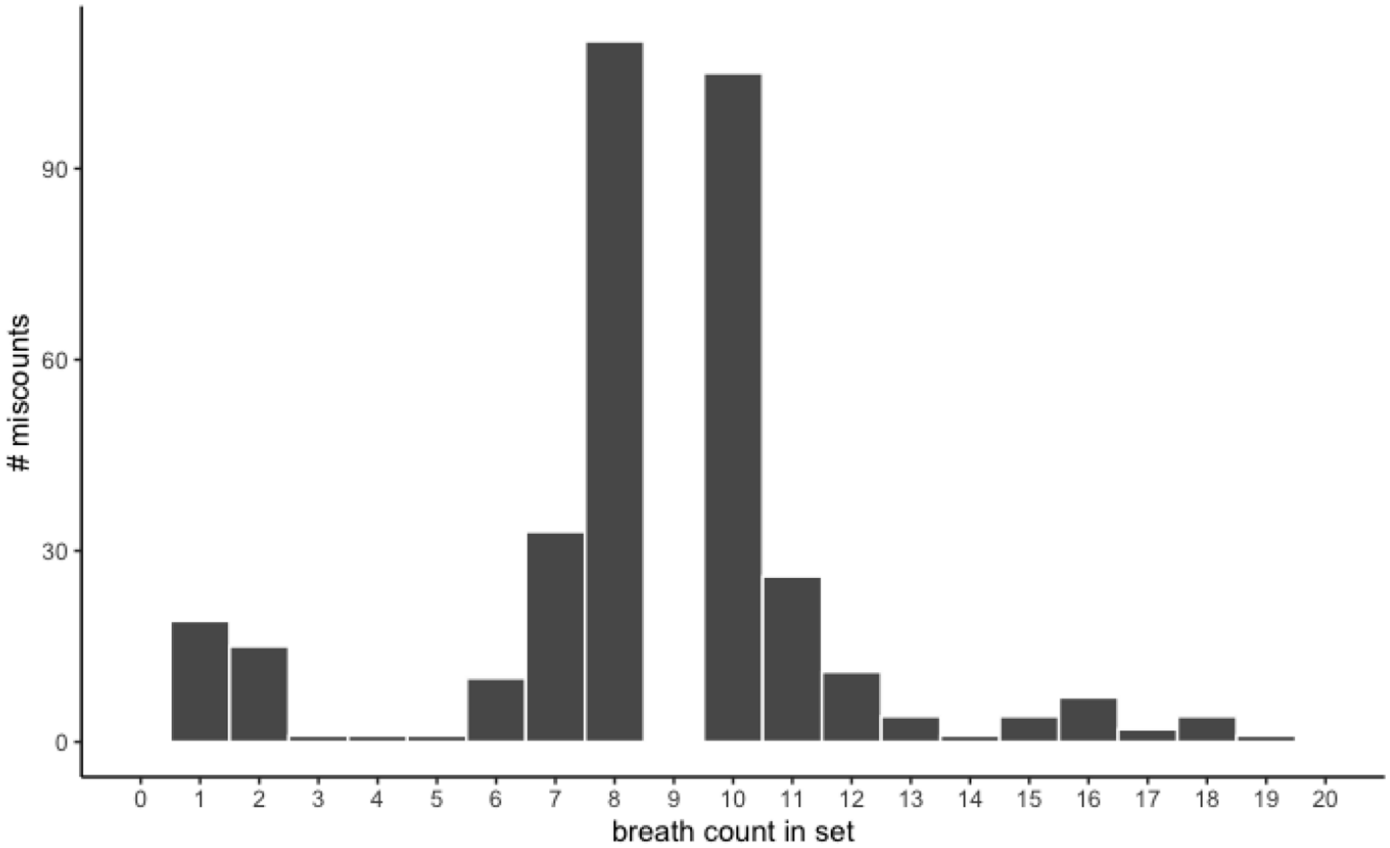
Miscounts by breaths counted in set. The majority of miscounts (pressing the up arrow to mark the ninth breath in a set) occurred at the eighth (33.07%) or 10th (28.99%) breath in a counted set. In other words, most miscounts occurred around the target 9th breath and suggests that children were mostly on task.

**Figure 3:**
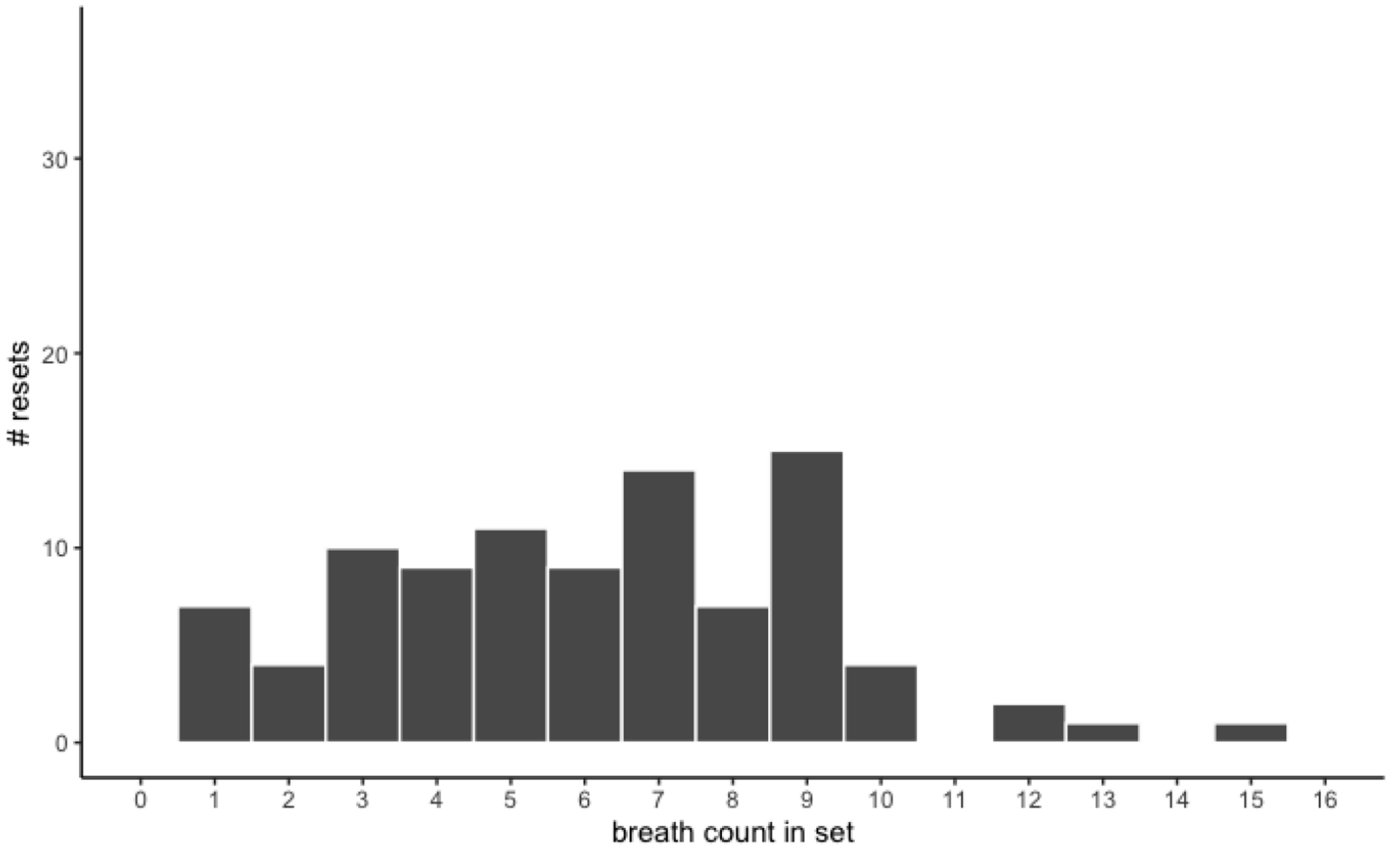
Resets by number of breaths counted in set. Resets were fairly evenly distributed across breath counts in a set prior to breath 9 and tapered off after breath 10, suggesting that children tended to lose count of their breath during a set.

Next, we examined the patterns of errors made. Following scoring procedures in adults, we accounted for two types of inaccurate sets: miscounts (any set ending in a down arrow press *without* eight preceding up arrow presses) and resets (any use of the reset spacebar to end a set). Children overall made more miscounts (67.6%) than resets (32.4%), and the percentage of miscounts and resets were negatively correlated with one another, *r* = −0.23, *p* = 0.02, such that those with more miscounts also had fewer resets overall. Closer examination of the counting patterns during miscounts and resets showed that most miscounts occurred around breath 9 (Figure 2) while resets were more evenly distributed across breaths in a set (Figure 3). This suggests that children were mostly on task as they made most errors during rather than after a designated nine-breath set, and miscounts were especially likely around the target 9th breath. These error patterns mostly align with those reported in adults (Clapper et al., 2021; Wong et al., 2018).

#### 3.1.2 Respiratory rate effects

We explored the possibility that children who breathed more slowly and counted fewer sets may also have had higher breath counting accuracy, because they had fewer opportunities to make mistakes. Conversely, it is also possible that children who counted fewer sets may have been more impacted by errors, because an error in fewer sets would count for a higher percentage loss in accuracy. We examined the correlation between counting accuracy and total sets to assess these possibilities and found no significant relationship between % accurate sets and the total sets counted, *p* = 0.88. This suggests that children’s respiratory rate is not related to their accuracy in breath counting.

#### 3.1.3 Time on task effects

Next, we examined the degree to which time on task might have impacted performance. We examined the occurrence of resets and miscounts by set number; the effects of set number on resets and miscounts were both non-significant, *p* = 0.117 and *p =* 0.505, respectively. Comparisons of the % resets and % miscounts between the first and second halves of the task were also non-significant, *p*’s > 0.86. Thus, time on task did not appear to affect children’s breath counting performance.

#### 3.1.4 Reliability of scores

To assess the internal reliability of breath counting indices, we calculated the split-half reliabilities of the three metrics from the BCT. Spearman-Brown corrected split-half reliabilities (calculated from 5,000 random split-half permutations) ranged between 0.39 and 0.55, reflecting poor to moderate internal reliabilities (Table 2). These values are very similar to those found in adults (Wong et al., 2018).

### 3.2 Breath counting and self-reported mindfulness

To assess the convergent validity of the breath counting task with self-reported mindfulness, we first examined pairwise correlations between breath counting indices and self-reported mindfulness scores. All pairwise correlations between self-reported mindfulness and breath counting indices were non-significant (*p*’s > 24; Table 3). However, because self-reported mindfulness measures might be prone to social desirability bias, we opted to statistically control for this bias to more directly examine relationships between breath counting and self-reported mindfulness. To capture self-reported mindfulness without the influence of social desirability bias, we first specified a latent factor model where summary scores from the three mindfulness questionnaires (MAAS-C, CAMM, and MICA) loaded onto a common mindfulness latent factor, and allowed covariances between each mindfulness score and the CSDS to remove variance due to social desirability bias from the self-reported mindfulness latent factor. Because this model was fully saturated, fit indices are unavailable; however, all factor loadings and covariances between mindfulness questionnaires and the CSDS were significant, all *p*’s < 0.03. We proceeded with this as our base model for subsequent analyses examining relationships between self-reported mindfulness and breath counting performance.

**Table 3:** Descriptives and correlations among mindfulness and breath counting indices.

Variable	*M*	SD	1	2	3	4	5	6	7
1. Age	11.64	1.12							
2. MAAS-C	4.14	0.81	−0.08						
3. CAMM	2.54	0.64	−0.05	0.70**					
4. MICA	4.02	0.46	−0.13	0.46**	0.37**				
5. CSDS	6.88	2.33	−0.14	0.36**	0.35**	0.24*			
6. Accuracy %	57.02	20.93	0.18	0.10	0.01	−0.01	−0.05		
7. Resets %	11.19	11.95	−0.01	0.04	0.05	0.01	−0.00	−0.34**	
8. Miscounts %	31.78	20.28	−0.18	−0.13	−0.04	0.00	0.06	−0.83**	−0.24*

None of the three breath counting indices (% accuracy, % resets, and % miscounts) significantly correlated with any of the child-reported mindfulness scores (MAAS-C, CAMM, MICA) nor social desirability bias (CSDS). *p < 0.05, **p < 0.01. MAAS-C, mindful attention awareness scale-children, CAMM, child and adolescent mindfulness measure; MICA, mindfulness inventory for children and adolescents; CSDS, children’s social desirability scale-short.

**Figure 4:**
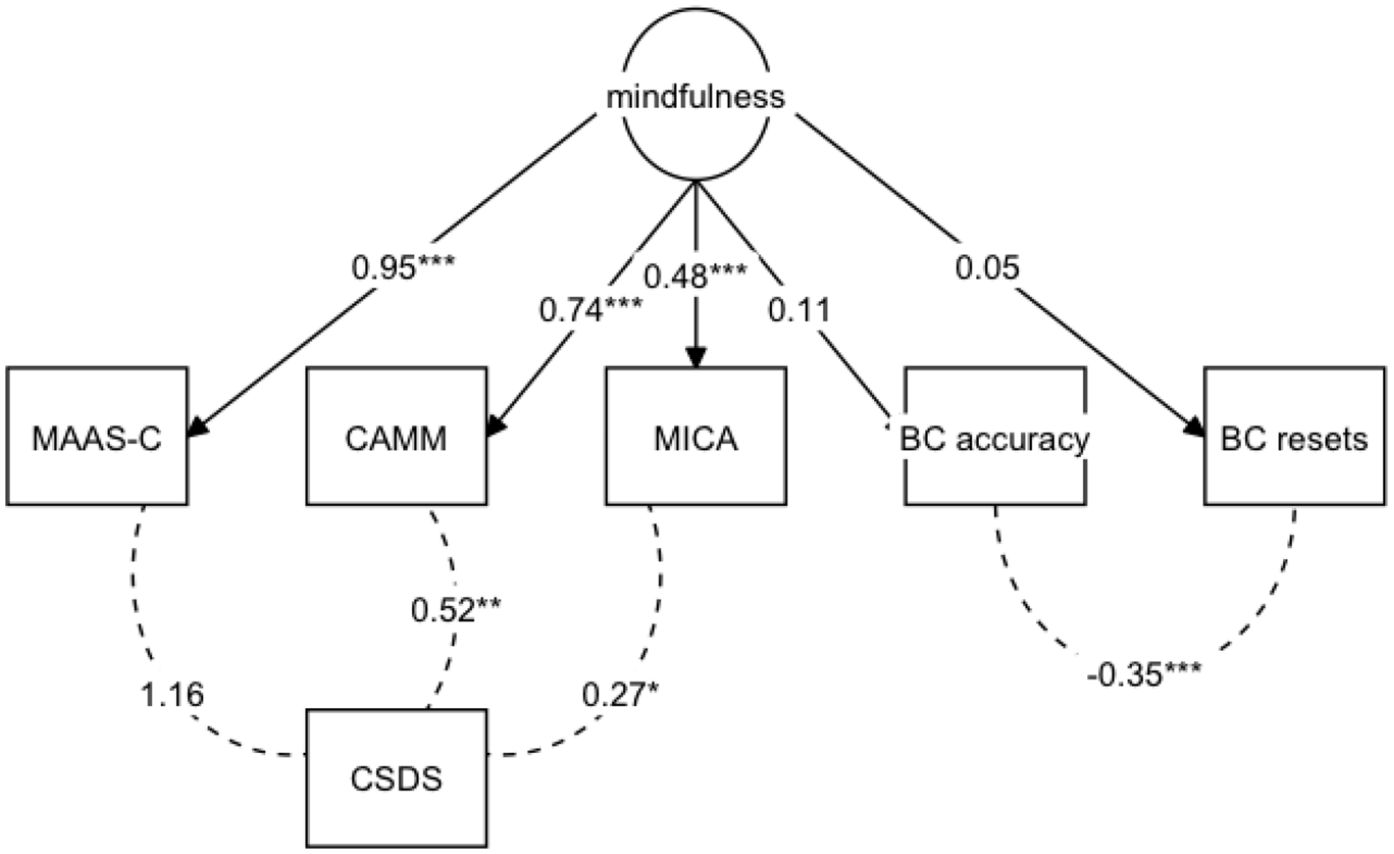
Mindfulness latent factor model with self-reported mindfulness and breath counting indices. Overall model fit is excellent, *χ*^2^(6) = 0.949, CFI = 1.000, TLI = 1.106, RMSEA = 0.000, SRMR = 0.021, but the estimated regression coefficients between the mindfulness latent factor and breath counting indices were nonsignificant, % accuracy *z* = 1.113, *p* = 0.266 and % resets *z* = 0.492, *p* = 0.622. Standardized estimates and their significances are shown. *p < 0.05, **p < 0.01. MAAS-C, mindful attention awareness scale-children; CAMM, child and adolescent mindfulness measure; MICA, mindfulness inventory for children and adolescents; CSDS, children’s social desirability scale-short; BC, breath counting task.

Next, to examine the relationship between breath counting performance and self-reported mindfulness after accounting for social desirability bias, we set the 3 breath counting indices—% accuracy, % miscounts, and % resets—to load onto the self-reported mindfulness latent factor from the base model (Supplementary Figure S1). The overall fit of this model was extremely poor, *χ*^2^(12) = 1750.723, *p* < 0.001, CFI = 0.053, TLI = −0.658, RMSEA = 1.235, SRMR = 0.157, and none of the breath counting indices loaded significantly onto the mindfulness latent factor, all *p’*s > 0.170. We suspected that given the high correlations among the breath counting indices (*r’s* > 0.54; Table 2), allowing all three breath counting indices to load onto the same factor may have caused the model to suffer from multicollinearity and resulted in misfit. Thus, we modified the model by removing % miscounts (which correlated most strongly with the main breath counting index, % accuracy, *r =* −0.83) and allowing the error terms of % accuracy and % resets to covary, to account for task-based variance. This modified model (Figure 4) showed excellent overall fit, *χ*^2^(6) = 1.651, *p* = 0.949, CFI = 1.000, TLI = 1.106, RMSEA = 0.000, SRMR = 0.021, but the estimated loadings between the mindfulness latent factor and breath counting % accuracy, *z* = 1.113, *p* = 0.266, and % resets, *z* = 0.492, *p* = 0.622, remained nonsignificant. Similar results were observed when we removed % resets and included only % accuracy, the main index from the breath counting task (Supplementary Figure S2): overall fit indices were excellent, *χ*^2^(3) = 1.431, CFI = 1.000, TLI = 1.055, RMSEA = 0.000, SRMR = 0.024, but % accuracy did not load significantly onto the self-reported mindfulness latent factor, *z* = 0.104, *p* = 0.270. All alternative model specifications (see Supplementary material S1) did not reveal any significant relationships between breath counting indices and self-reported mindfulness. All together, these results suggest children’s breath counting performance did not demonstrate convergent validity with self-reported mindfulness, even after controlling for social desirability bias.

### 3.3 Breath counting and cognitive control

Next, we examined children’s behavioral cognitive control and its associations with breath counting performance and self-reported mindfulness. To begin, we first examined children’s performance patterns in the CTS. A linear mixed effects model predicting *z*-scored reaction times by CTS condition (Pro-Imp vs. Pro-Poss) revealed a significant condition effect, *b* = −0.749, SE = 0.043, *t* = −17.49, *p* < 0.001, *d* = 0.847, *η*^2^ = 0.152, where reaction times in the Pro-Poss condition were significantly faster than in the Pro-Imp condition. The condition effect in this model was allowed to vary between individuals; these individually estimated condition effects reflected the degree to which each individual’s response times decreased in the Pro-Poss condition compared to the Pro-Imp condition and served as individuals’ zRT slope scores in subsequent analyses. These zRT slopes were largely negative in our sample (*M =* −0.41, SD *=* 0.09), reflecting overall faster reaction times in the Pro-Poss condition and suggesting that most children adaptively engaged proactive control when it was made possible. No significant condition effect was observed in a comparable logistic mixed effects model predicting accuracies by condition, *p* = 0.217. Overall, these findings suggest that children exhibited faster reaction times in the Proactive-Possible compared to the Proactive-Impossible conditions of the CTS, reflecting their engagement of proactive control when possible.

Next, we examined relationships between children’s CTS and breath counting performance indices. Correlations between performance indices from the CTS and breath counting are shown in Table 4. We observed significant pairwise correlations between breath counting % accuracy and CTS mean accuracy, *r* = 0.259, *p* = 0.012, and between breath counting % accuracy and CTS median reaction times, *r =* −0.305, *p* = 0.003, such that those who were more accurate and faster on the CTS also showed higher % accuracy in the breath counting task. The association between breath counting accuracies and CTS median reaction times was not driven by performance in a particular condition of the CTS, as reaction times in both CTS conditions correlated significantly with breath counting accuracies, *p’s* < 0.007, and a Fisher’s z-test showed that these correlations were not significantly different from one another, *z* = 1.508, *p* = 0.132 (see Table 4 for correlations). The correlations between breath counting accuracies and CTS accuracies also did not differ significantly between the Pro-Poss and Pro-Imp conditions, *z* = −0.578, *p* = 0.563; however, the pairwise correlation between mean accuracies in the Pro-Poss condition and breath counting accuracies was marginal, *r* = 0.19, *p* = 0.07. No significant relationships were found between breath counting indices and any of the other CTS measures we examined, including mean combined switch costs (task switching), zRT slopes (preference for proactive control), and total double errors (attentional lapses), all *p’s* > 0.07 (Table 4). We also did not observe any significant correlations between CTS performance and self-reported mindfulness scores, all *p’s* > 0.20. Overall, these findings suggest that children who performed well under both Pro-Poss and Pro-Imp conditions in the CTS also demonstrated higher breath counting accuracy, and that the adaptive engagement of proactive control was not associated with breath counting performance in our sample.

**Table 4:** Correlations among cued task switching (CTS) and breath counting task (BCT) scores.

Variable	*M*	SD	1	2	3	4	5	6	7	8	9	10	11
1. CTS: ACC, mean	0.87	0.1											
2. CTS: ACC mean ProPoss only	0.87	0.1	0.84**										
3. CTS: ACC mean ProImp only	0.87	0.12	0.89**	0.50**									
4. CTS: RT, median	1.1	0.33	0.09	−0.02	0.17								
5. CTS: RT median ProPoss only	0.86	0.35	0.02	−0.06	0.08	0.94**							
6. CTS: RT median ProImp only	1.31	0.36	0.03	−0.04	0.09	0.93**	0.82**						
7. CTS: combined switch costs	282.7	56.45	−0.86**	−0.75**	−0.75**	−0.21*	−0.14	−0.16					
8. CTS: zRT slope	−0.41	0.09	−0.02	−0.05	0.01	0.25*	0.42**	−0.04	0.02				
9. CTS: double errors, sum	2.46	4.69	−0.91**	−0.73**	−0.84**	−0.08	0	−0.03	0.75**	0.04			
10. BCT: % resets	11.19	11.95	−0.11	−0.05	−0.13	−0.1	−0.12	−0.04	0.17	−0.18	0.04		
11. BCT: % accuracy	57.02	20.93	0.26*	0.19	0.25*	−0.31**	−0.28**	−0.37**	−0.19	0.04	−0.18	−0.34**	
12. BCT: % miscounts	31.78	20.28	−0.21*	−0.17	−0.19	0.38**	0.36**	0.41**	0.1	0.06	0.16	−0.24*	−0.83**

Breath counting % accuracy and % miscounts showed significant correlations with cued task switching mean accuracy and median reaction times, *p*’s < 0.05. All other pairwise relationships were non-significant. *p < 0.05, **p < 0.01. CTS, cued task switching; ACC, accuracy; RT, reaction time; BCT, breath counting task.

### 3.4 Changes after 1–2-week mindfulness practice

**Figure 5:**
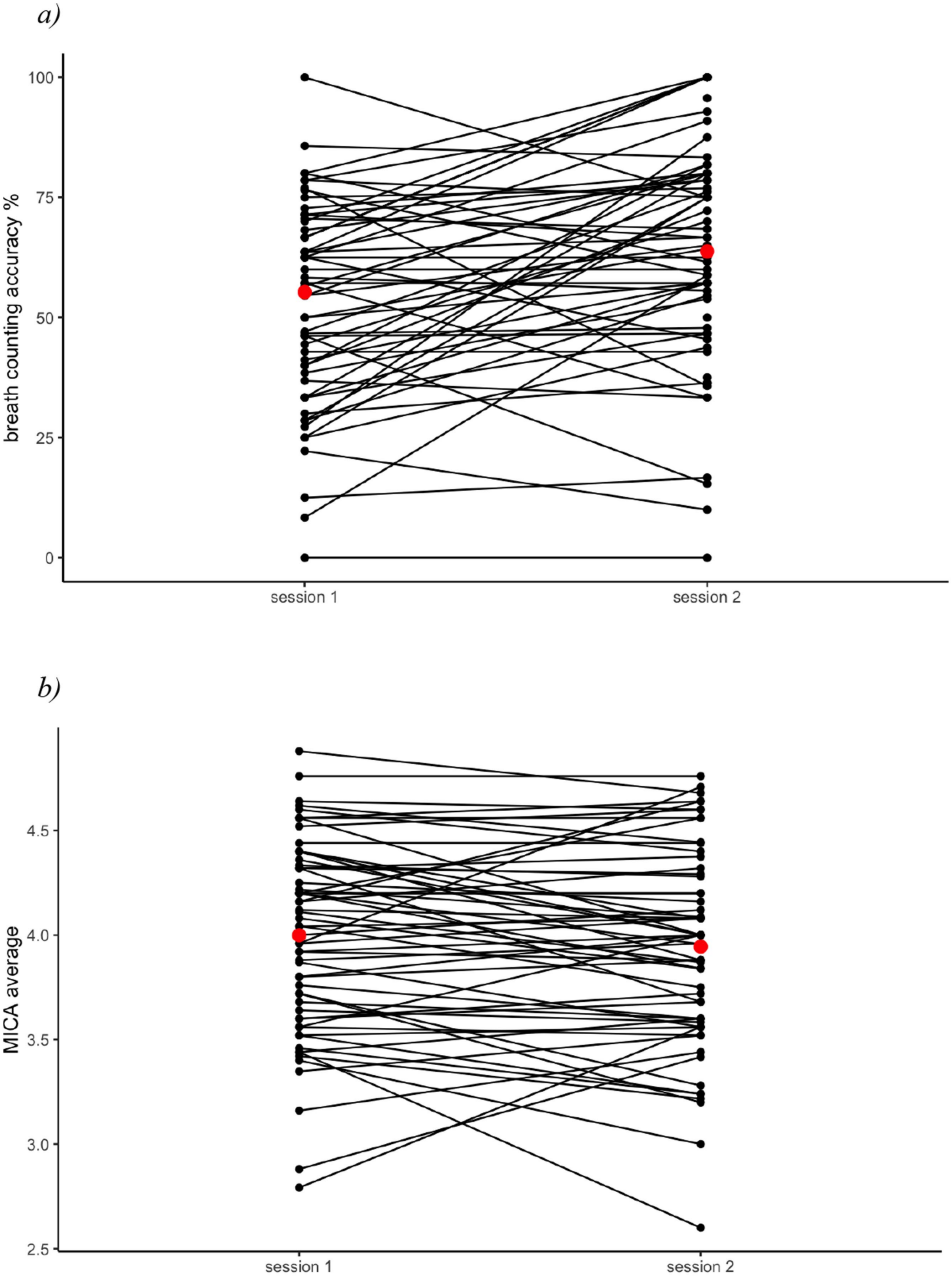
Scores pre and post 1–2 week mindfulness practice. **(a)** Change in breath counting accuracy. **(b)** Change in MICA average scores. After completing 1–2 weeks of daily mindfulness practice, children significantly improved in their breath counting performance, *p* < 0.001, but did not change in their MICA scores, *p* = 0.44. Red dots indicate the session mean.

In the subset of children who completed the 1–2-week daily mindfulness practice and returned for a second session (*n =* 67), we examined changes in their mindfulness as measured by the MICA and the breath counting task. We found no significant differences in their MICA scores between sessions, *t*(66) = 0.77, *p* = 0.44 (Figure 5a), but significantly higher breath counting accuracy at session 2 than session 1, *t*(56) = 4.05, *p* < 0.001, *d* = 1.08, *η*^2^ = 0.23 (Figure 5b). This improvement was not predicted by the number of daily mindfulness sessions that children completed, *t* = 1.056, *p* = 0.30, but this absence of a dosage effect might have been due to the limited range of sessions completed, as most children completed 6–9 sessions (62.3%). Improvements in breath counting accuracy were greater in children with lower breath counting accuracies at session 1, *t* = −3.595, *p* < 0.001. Altogether, these results suggest that 1–2 weeks of daily mindfulness practice predicted increases in children’s breath counting accuracy but not in their self-reported mindfulness scores. Thus, the breath counting task might be a more sensitive and valid index of improvements following mindfulness practice compared to self-report measures in children.

## 4 Discussion

Expanding our toolbox of mindfulness measures is crucial for furthering progress in the field of mindfulness research, especially among child populations. In the current study, we examined the validity of a behavioral measure of mindfulness, the breath counting task, for children ages 9–13 years. Our results showed that breath counting patterns in children were similar to those in adults, and that children with higher accuracy in the breath counting task also showed faster speed and marginally better accuracy in the cued task switching paradigm, a performance-based measure of cognitive control. Children’s breath counting performance was unrelated to their self-reported mindfulness but, unlike self-reported mindfulness, improved significantly after 1–2 weeks of daily breath-focused mindfulness practice. These results suggest that although breath counting performance did not show convergent validity with self-reported mindfulness measures in children, it was associated with cognitive control and responsive to recent mindfulness practice, suggesting compelling reasons to consider its use when measuring mindfulness in children.

Contrary to our hypothesis, we did not find that children’s self-reported mindfulness predicted their breath counting task performance. This diverges from studies in adults that have identified significant, albeit weak, correlations between breath counting accuracy and self-reported mindfulness measures (Levinson et al., 2014; Wong et al., 2018), but aligns with recent similar psychometric investigations in ruminative adolescents (Treves et al., 2025). More broadly, the absence of a significant correlation mirrors patterns observed in other areas of psychology, where self-report and objective measures of complex constructs often show weak or negligible associations with one another (e.g., Saunders et al., 2018). In the following we consider three possible explanations for this null finding. First, it is possible that a correlation exists, but we were underpowered to detect it. Previous findings in adults revealed only weak relationships between breath counting accuracies and self-reported mindfulness, with Pearson’s *r*’s ranging from 0.164 (Wong et al., 2018) to 0.20 (Levinson et al., 2014); findings are also mixed as non-significant relationships have also been reported (Isbel et al., 2020). Assuming that a weak true population correlation of *r* = 0.20 exists between breath counting accuracy and reported mindfulness scores in children (based on Levinson et al., 2014’s findings in adults), our final sample size of 95 participants only had a power of 0.62 to detect this correlation (at an alpha level of 0.05). Further, due to the noisier nature of measures obtained from child participants, our power may have been further attenuated. To explore this possibility, future work should attempt to replicate this study with a larger sample to determine whether a weak correlation might exist between children’s mindfulness and breath counting performance.

A second possibility might be that self-report mindfulness questionnaires do not accurately measure mindfulness in children. Self-reported questionnaires are prone to social desirability bias, as indicated by the significant associations we found between the Children’s Social Desirability Scale and the mindfulness questionnaires in our sample, and assume a level of metacognitive awareness that may not be reasonable for children. Further, items in self-report mindfulness questionnaires tend to focus on more abstract aspects of mindfulness such as self-acceptance or metacognitive awareness (e.g., “I do not blame myself if I make a mistake,” MICA item 11; “I cannot stop thinking about the past or the future,” MAAS-C item 13), but mindfulness activities for children tend to focus more on present-moment awareness (Vekety et al., 2022). Some self-reported items attempt to capture present-moment aspects of mindfulness (e.g., “It is hard for me to pay attention to only one thing at a time,” CAMM item 6), they still require a degree of metacognitive awareness to accurately respond to; this can be demanding on children and increase measurement error in their responses. Given these considerations, it is possible that children’s mindfulness is more accurately captured by task-based measures, such as the breath counting task, than by self-reports. Future work will be needed to further investigate the validity of self-report measures in accurately assessing mindfulness in children.

Finally, it is also possible that children’s breath counting performance does not accurately or comprehensively measure their mindfulness. Indeed, our findings suggest that children’s breath counting performance more closely reflects cognitive processes, such as sustained attention or cognitive control, than their self-reported mindfulness. Previous studies in adults have identified attentional maintenance as a key cognitive component that supports breath counting accuracy (Levinson et al., 2014; Wong et al., 2018), and one study of ruminative adolescents also identified a significant correlation between attentional maintenance and breath counting resets (Treves et al., 2025). Similarly, our results showed that children’s overall performance in cued task switching, a task that requires attentional maintenance and cognitive control, is positively associated with their breath counting accuracy. These findings suggest that cognitive control may play a key role in children’s breath counting performance, perhaps due to the breath counting task’s demands on attentional maintenance required to stay on task over several minutes. Because cognitive control develops gradually across childhood (Munakata et al., 2012; Crone and Steinbeis, 2017; Davidson et al., 2006), the breath counting task might place a heavy demand on children’s developing cognitive control resources and thus be more closely related to children’s cognitive processes than their mindfulness per se. Future work should examine how the relationships among breath counting, mindfulness, and cognitive control change across development, and work to determine at which stage of development the breath counting task begins showing convergent validity with self-reported mindfulness.

It remains unclear, however, the mechanisms by which cognitive control is engaged during breath counting, and how their association connects to mindfulness more broadly. Contrary to our initial hypothesis, our results showed that more adaptive engagement of proactive control was not associated with breath counting performance in children. This absence of an effect may have been due to the limited range of zRT slopes in our sample, as almost all children displayed faster reaction times in the proactive-possible compared to the proactive-impossible condition and thus sported negative zRT slopes, reducing our power to detect an effect. Alternatively, it is also possible that adapting proactive control is not by itself relevant for breath counting performance. As our findings indicated, children’s overall performance in the Cued Task Switching paradigm, in both Proactive-Possible and Proactive-Impossible conditions, was positively associated with their breath counting accuracy. This suggests that both proactive and reactive control, or overall performance more generally, may be relevant for breath counting performance. This interpretation aligns with some work in adults suggesting that those with higher self-reported mindfulness demonstrated more balanced, flexible use of proactive and reactive control (Chang et al., 2018; Aguerre et al., 2021). These findings, together with ours, lend support to the theory that mindfulness encourages non-attachment to preferences for one mode of cognitive control over another and thus more flexibility to engage either form of cognitive control when needed (Aguerre et al., 2021). However, based on current findings it is unclear whether more mindful individuals are better at both reactive and proactive control, simply do not hold a preference for one mode of control, or are just overall better at performing cognitive tasks. Further work is needed to clarify the relationships between cognitive control and mindfulness, especially as measured by the breath counting task, in order to more clearly understand how the two modes of cognitive control interact with mindfulness.

One might ask, given the lack of convergent validity between self-reported mindfulness and breath counting measures, should researchers avoid using self-reports or the breath counting task to assess children’s mindfulness skills? We do not believe this to be the case, but rather, argue that both types of measures can tap different aspects of mindfulness abilities. Indeed, our finding that after 1–2 weeks of daily breath-focused mindfulness practice children significantly improved in their breath counting accuracies but not in their self-reported mindfulness suggests that the breath counting task might be better able to detect immediate changes after recent mindfulness practice than self-reported measures. This finding aligns with those from adults showing that breath counting performance improved specifically after mindfulness training while self-report measures improved indiscriminately in both the training and control groups (Isbel et al., 2020). Together, these findings further support the discriminant validity of the breath counting task compared to self-reports.

One framework to conceptualize this difference is to consider mindfulness in state and trait forms (Bravo et al., 2018). The breath counting task might be more suited to capturing state mindfulness related to present-moment attention and awareness, while self-reports capture trait mindfulness related to non-judgment and metacognition. Both measures may speak to different but related aspects of mindfulness, and both may add value to our understanding of mindfulness at multiple levels of this construct. Future work with children and adults should consider incorporating both self-report and behavioral measures of mindfulness, such as the breath counting task, in order to more comprehensively capture this complex construct.

We acknowledge several limitations in the current study and provide recommendations to build upon our work. First, our sample size (*N =* 109) is sufficient for several regression analyses but underpowered to assess the fit of complex latent factor models—future work should attempt to replicate and supplement our findings with larger sample sizes to further explore possible relationships between breath counting and mindfulness in children. Second, with regards to the mindfulness intervention, our study employed a single-group pre-test post-test intervention design, which does not support strong causal inferences. Due to the lack of a control group, we cannot rule out alternative explanations for the improvements in breath-counting following training in our sample, such as test–retest effects or the passage of time. Future studies employing control groups are needed to disentangle the intervention’s effects from these alternative explanations. Third, because the daily mindfulness practice sessions focused on breath awareness, it is conceivable that improvements in children’s breath counting were simply due to their practicing breathing exercises and not necessarily mindfulness per se. Future work should seek to untangle the multidimensionality of mindfulness and how it changes across practice and experience, using self-report and behavioral measures, in children and adults.

Overall, the current findings suggest that in children ages 9–13 years, the breath counting task may capture aspects of mindfulness separate from those captured by self-reports. Breath counting performance was related to children’s overall cognitive control performance, thus pointing to a potential cognitive mechanism that underlies breath awareness and mindfulness in children. Further, breath counting performance improved after 1–2 weeks of daily mindfulness exercises while self-reported mindfulness did not, suggesting that the breath counting task is more sensitive to immediate effects of mindfulness practice than self-reports in children. We encourage future investigations to replicate and expand upon our findings to better understand how breath counting, cognitive control, and self-reported mindfulness interact to support children’s wellbeing.

## Data Availability

The datasets presented in this study can be found in online repositories. The names of the repository/repositories and accession number(s) can be found at: https://osf.io/47tbw/?view_only=cc6c63772d934088b3f14ebf165218ff.

## Footnotes

^1^ https://osf.io/4cnue/
