# Correction: Measuring mindfulness in children: breath counting is unrelated to self-reported mindfulness but improves after mindfulness practice in 9–13 year-olds

**DOI:** 10.3389/fpsyg.2025.1740311

**Published:** 2025-11-20

**Authors:** Winnie Zhuang, Laura E. Michaelson, Sona Dimidjian, Yuko Munakata

**Affiliations:** 1 Renée Crown Wellness Institute, University of Colorado Boulder, Boulder, CO, United States; 2 Department of Psychology and Center for Mind and Brain, University of California, Davis, Davis, CA, United States; 3 American Institutes for Research, Arlington, VA, United States; 4 Department of Psychology and Neuroscience, University of Colorado Boulder, Boulder, CO, United States

**Keywords:** mindfulness, self-report, breath counting task, cognitive control, children

Affiliation “Department of Psychology and Center for Mind and Brain, University of California, Davis, Davis, CA, United States” was omitted for author Winnie Zhuang. This affiliation has now been added for author Winnie Zhuang.

In the **Acknowledgments**, this project began at the University of Colorado Boulder and support from the UC Davis Open Access Fund were erroneously omitted.

The original version of this article has been updated.

