# Supplementary material for: Measuring mindfulness in children: breath counting is unrelated to self-reported mindfulness but improves after mindfulness practice in 9–13 year-olds
Source: Front Psychol. 2025 Sep 30;16:1644127. doi: 10.3389/fpsyg.2025.1644127 (PMC12518087; doi:10.3389/fpsyg.2025.1644127)
Supplement: Supplementary file 1 [file Data_Sheet_1.PDF]

Supplementary Materials

S1. Alternative models exploring the relationship between breath counting and self-reported mindfulness

We estimated several alternative model specifications to explore the relationship between the breath counting task and self-reported mindfulness and present them in Figures S1 - S3 below. None of these alternative models revealed any significant associations between breath counting indices and self-reported mindfulness questionnaires.

Figure S1

Mindfulness latent factor with all 3 breath counting indices

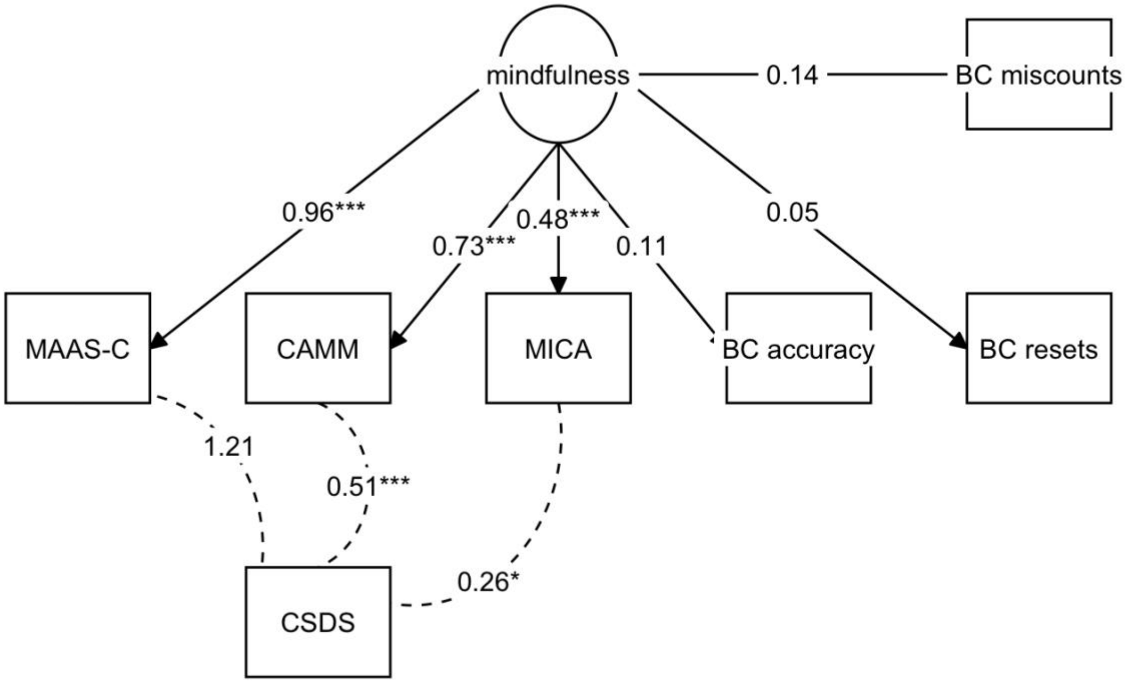

Notes. In this model, we allowed all three breath counting indices to load onto the mindfulness latent factor. The overall fit of this model was extremely poor, $\chi^2(12) = 1750.723$, $p < .001$, CFI = 0.053, TLI = -0.658, RMSEA = 1.235, SRMR = .157, and none of the breath counting indices loaded significantly onto the mindfulness latent factor, all *p'*s > .170. Standardized estimates and their significances are shown. * indicates $p < .05$. ** indicates $p < .01$. *Abbreviations*: MAAS-C = Mindful Attention Awareness Scale–Children, CAMM = Child and Adolescent Mindfulness Measure, MICA = Mindfulness Inventory for Children and Adolescents, CSDS = Children's Social Desirability Scale–Short, BC = breath counting task.

Figure S2

Mindfulness latent factor with breath counting accuracy only

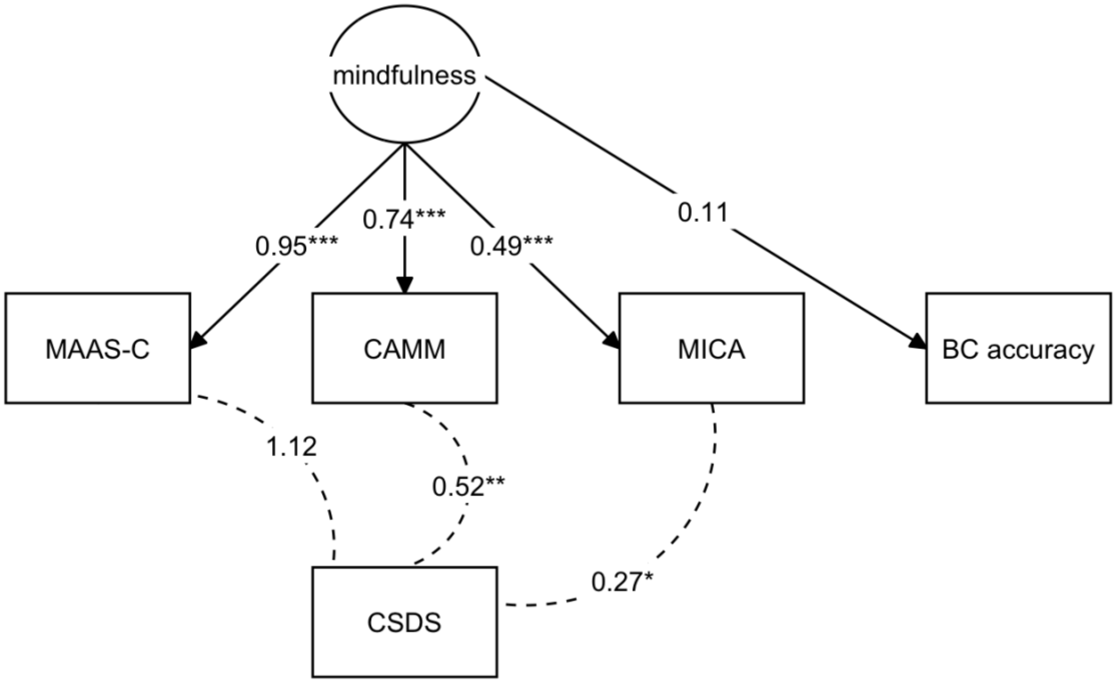

Notes. In this model, we included only % accuracy, the main index from the breath counting task and allowed it to load onto the mindfulness latent factor. Overall fit indices were excellent, $\chi^2(3) = 1.431$, CFI = 1.000, TLI = 1.055, RMSEA = .000, SRMR = .024, but **breath counting** % accuracy did not load significantly onto the self-reported mindfulness latent factor, $z = .104$, $p = .270$**.** Standardized estimates and their significances are shown. * indicates $p < .05$. ** indicates $p < .01$. *Abbreviations*: MAAS-C = Mindful Attention Awareness Scale–Children, CAMM = Child and Adolescent Mindfulness Measure, MICA = Mindfulness Inventory for Children and Adolescents, CSDS = Children's Social Desirability Scale–Short, BC = breath counting task.

Figure S3

Alternative 2-factor model with separate self-reported mindfulness and breath counting task latent factors

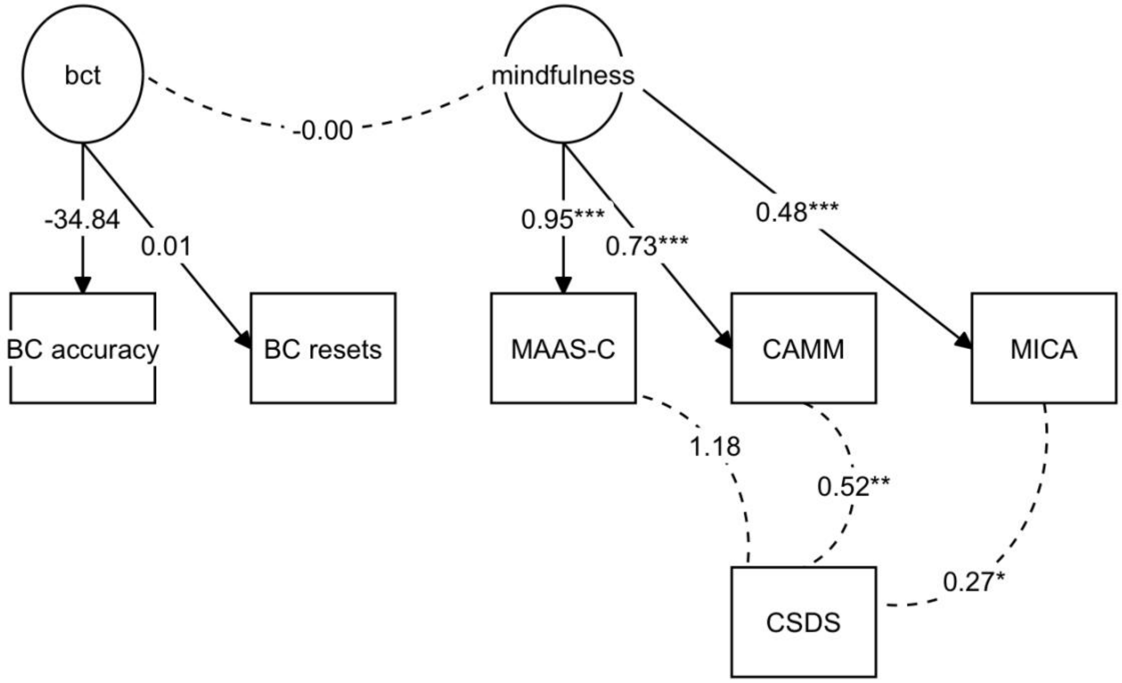

Note. This alternative 2-factor model with separate breath counting and self-reported mindfulness latent factors showed overall excellent fit, $\chi^2(6) = 1.895$, $p = .929$, CFI = 1.000, TLI = 1.100, RMSEA = 0.000, SRMR = 0.027, but a non-significant covariance between the mindfulness and breath counting latent factors, z = -.885, $p = .376$. Further, breath counting % accuracy and % resets did not load significantly onto the breath counting latent factor, *p*'s>.24, and the estimates for their factor loadings are suggestive of a Heywood case, likely due to model misspecification (Kolenikov & Bollen, 2012; Farooq 2022). Fixing the breath counting loadings to equal did not improve fit. Standardized estimates and their significances are shown. * indicates $p < .05$. ** indicates $p < .01$. *Abbreviations*: MAAS-C = Mindful Attention Awareness

Scale–Children, CAMM = Child and Adolescent Mindfulness Measure, MICA = Mindfulness Inventory for Children and Adolescents, CSDS = Children's Social Desirability Scale–Short, BC = breath counting task.
